# Estimation of Beta-Blocker Consumption in the Urban Population Using the Wastewater-Based Epidemiology Approach

**DOI:** 10.3390/molecules31081249

**Published:** 2026-04-09

**Authors:** Mihail Simion Beldean-Galea, Mihaela-Cătălina Herghelegiu, Ye Yang, Robert Tötös, Maria Concetta Bruzzoniti, Ioana Elena Beldean-Galea, Sorina Irimie, Anda Curta

**Affiliations:** 1 Faculty of Environmental Science and Engineering, Babeș-Bolyai University, 30 Fântânele Str., RO-400294 Cluj-Napoca, Romania; ye.yang@ubbcluj.ro; 2 “Raluca Ripan” Institute for Research in Chemistry, Babeș-Bolyai University, 30 Fântânele Str., RO-400294 Cluj-Napoca, Romania; 3 Faculty of Chemistry and Chemical Engineering, Babeş-Bolyai University, 11 Árány Janos Str., RO-400028 Cluj-Napoca, Romania; robert.totos@ubbcluj.ro; 4 Department of Chemistry, University of Turin, Via P. Giuria 5, 10125 Turin, Italy; mariaconcetta.bruzzoniti@unito.it; 5 National Institute of Public Health, Regional Centre of Public Health Cluj, 6 Pasteur Str., RO-400349 Cluj-Napoca, Romania; ioana.beldean@insp.gov.ro (I.E.B.-G.); sorina.irimie@insp.gov.ro (S.I.); anda.curta@insp.gov.ro (A.C.)

**Keywords:** wastewater-based epidemiology, beta-blockers, human consumption, solid phase extraction, liquid chromatography coupled with tandem mass spectrometry

## Abstract

Wastewater-based epidemiology (WBE) is an approach that uses information obtained from the analysis of various metabolites or residues in wastewater with the aim of assessing the consumption of or exposure to chemicals or pathogens in a population connected to a sewage system. The aim of this work was to develop methods for the isolation and analysis of seven beta-blockers (acebutolol, atenolol, betaxolol, metoprolol, nadolol, pindolol and propranolol) in wastewater samples collected from the influent of the wastewater treatment plant in Cluj-Napoca, Romania, in order to estimate their consumption among the population in two time periods (February and October 2024) using WBE. The selected beta-blockers were extracted by solid phase extraction using a Strata C18-U cartridge and analyzed by liquid chromatography coupled with tandem mass spectrometry. The consumption was estimated using the daily mass load of pharmaceutical products reported per 1000 inhabitants (mg/day/1000inh) and varied in the following ranges: atenolol 0.03–3.74, nadolol 0.03–0.1, propranolol 0.04–0.72, betaxolol 0.07–0.38, and metoprolol 54.85–276.45. From the obtained results, it can be observed that metoprolol is the most used beta-blocker in the investigated population, followed by atenolol, propranolol and betaxolol. Other beta-blockers are consumed in small quantities or occasionally.

## 1. Introduction

Imbalances in the normal functioning of the human body most often require drug treatments, in order to remedy conditions and increase quality of life and longevity. Beta-blocking drugs (β-blockers or β-adrenergic antagonists) are the first-line drugs for cardiovascular conditions and represent the basic therapy in the treatment of heart disease, especially heart failure, acute myocardial infarction, hypertension, angina pectoris, and arrhythmias, but also anxiety, panic attacks, migraine, and hyperthyroidism, as well as topical medication for open-angle glaucoma [1,2,3,4]. The mode of action of these drugs is to block the body’s β-adrenergic receptors (β1, β2, β3), slowing down nerve impulses to the heart, and reducing the heart rate, thus protecting the body from the harmful effects of adrenaline [4,5]. First-generation beta-blockers are non-selective, acting on β1 and β2 receptors (propranolol, pindolol, and nadolol), while second-generation beta-blockers have a higher affinity for β1 receptors (atenolol, metoprolol, and acebutolol), and third-generation beta-blockers have selectivity for β3 receptors and varying affinity for β1 receptors (betaxolol) [5]. Due to their calming effects, beta-blockers can be abused to alleviate anxiety states, being present in the case of doping in athletes [6,7], in the prevention of stress and death of animals on their way to slaughterhouses [8] or in the case of horse racing [9]. Moreover, as a consequence of the increasing number of people suffering from cardiovascular diseases, the annual consumption of beta-blockers has been steadily increasing [10]. Atenolol, propranolol, metoprolol, bisoprolol and betaxolol are the most used beta-blockers [11]. In 2020, in Romania, 55.2% of deaths were caused by cardiovascular diseases [12].

The increase in the consumption of beta-blockers has led to an increase in the release of their metabolites into the environment, especially into water, due to their improper management, treatment and disposal [8,13]. Beta-blockers are considered pseudo-persistent pollutants, with half-lives in water between 0.4 and 13.1 ± 0.9 days [8,14]. Maszkowska et al. [15] showed that beta-blockers affect testosterone levels in male organisms, similarly to endocrine disruptors. Chronic or acute exposure of environmental organisms to drugs causes negative effects associated with their growth, reproduction and even death [8,16]. In the case of algae (*Raphidocelis subcapitata*, *Desmodesmus subspicatus*, and *Cyclotella meneghiniana*), beta-blockers inhibit growth, cause the death of fish larvae (*Danio rerio* and *Oryzias latipes*), and in *Danio rerio*, delayed hatching, deformities and slowed growth are also observed [16]. Propranolol increases the heart rate of *Daphnia magna* [17]. Atenolol decreases hemoglobin levels and, implicitly, oxygen levels in the gills of the catfish (*Oncorhynchus mykiss*) [18]. Metoprolol inhibits the growth of Nile tilapia (*Oreochromis niloticus*) and exhibits estrogenic activity at high concentrations [19].

Due to potential effects on environmental factors, one beta-blocker, namely propranolol, has been included on the European Union watch list [20], requiring its monitoring in water bodies. An important factor in monitoring and managing public health is knowledge of the use of these pharmaceuticals among the population.

A rapid methodology that offers the possibility of estimating drug use among a population is wastewater-based epidemiology [21]. This methodology represents an objective approach used to estimate the consumption of or exposure to chemicals or pathogens in a population connected to a sewage system that is based on the quantification of specific metabolites in wastewater samples from the influent of a wastewater treatment plant [21,22,23,24]. The measured concentrations of metabolites (ng/L) are converted to mass loads, by multiplying them with the daily wastewater flow rate (L/day), followed by the correction for the specific pharmacokinetics of the compounds and the normalization of these quantities with the population size present in the investigated catchment area (mg/day/1000 inhabitants) [24,25]. Expressing the quantities of investigated compounds in mg/d/1000inh is necessary to standardize the reporting of data per 1000 inhabitants, in this way, the population size variable is practically eliminated, and thus allows the comparison of the results with other results obtained in different geographical regions [26].

WBE was first applied two decades ago to estimate cocaine use in a population by analyzing residues from the Po River and sewage treatment plants [22] and, since its first application in 2005, the WBE method has developed rapidly to monitor temporal and spatial trends in substance use, especially illicit drugs [22,27,28], alcohol [29], nicotine [30,31] caffeine [32], pharmaceuticals [24,33,34,35,36], beta-blockers [6,37], and hormones [38], as well as other classes of compounds—sweeteners [39], pesticides [40], phthalate esters, bisphenols and terephthalic acid [41,42], infectious agents (viral and bacterial [43,44,45] or the health status of the population—the population’s diet [46], as well as the form of the body’s response to various internal processes (specific disease- or exposure-related proteins, genes and metabolites) [47,48].

This study aims to investigate the differences in beta-blocker consumption in two different seasons (winter and autumn) in Cluj-Napoca, Romania, using the WBE approach, thus providing effective information for the prevention and control of drug consumption and informing authorities about the health status of the target population.

## 2. Results

### 2.1. Estimating Population Size

The population size connected to the sewage system was calculated based on NH_4_-N, as the following values: 439,233 inhabitants for February 2024 and 532,261 inhabitants for October 2024, respectively [36] (Table S1). This estimate is very close to the real situation since in February the number of inhabitants is lower due to the exam period, or various inter-semester holidays, while in October the population is higher due to the start of the school year, which brings a substantial influx of students to the city.

### 2.2. Occurrence of Beta-Blockers in the Influent of Cluj-Napoca WWTP

Target metabolites were detected in all seven samples (n = 7) at variable frequencies and concentrations, ranging from not detected to 124.59 ng/L (Table S2). In February 2024, the presence of atenolol, betaxolol, metoprolol and propranolol was detected seven times (n = 7), nadolol four times (n = 4) and acebutolol and pindolol once (n = 1). In October 2024, atenolol, metoprolol, and propranolol were present in all samples (n = 7), betaxolol in four (n = 4), and nadolol in one (n= 1), while acebutolol and pindolol were not detected (n = 0). The minimum and maximum values of the monitored compounds are presented in Table 1.

The two months were selected due to the different consumption of beta-blockers as well as the different temperatures in winter and autumn. In autumn (October) the risk of cardiovascular diseases is lower than in winter (February) due to the higher temperature. Beta-blocker treatment in winter is necessary to ensure the oxygen requirement and maintain a constant body temperature due to the low temperature. Therefore, we can deduce that the consumption of beta-blockers is higher in winter. Moreover, the difference in environmental conditions can be another reason. High temperature, strong solar radiation, and variation in the sewer conditions (pH, biofilm) in autumn may trigger higher transformation rates and therefore reduce the levels of beta-blockers in the wastewater, thus explaining the differences in concentrations between the two investigated seasons, and between days (Table S2) [8,37,49].

**Figure 1:**
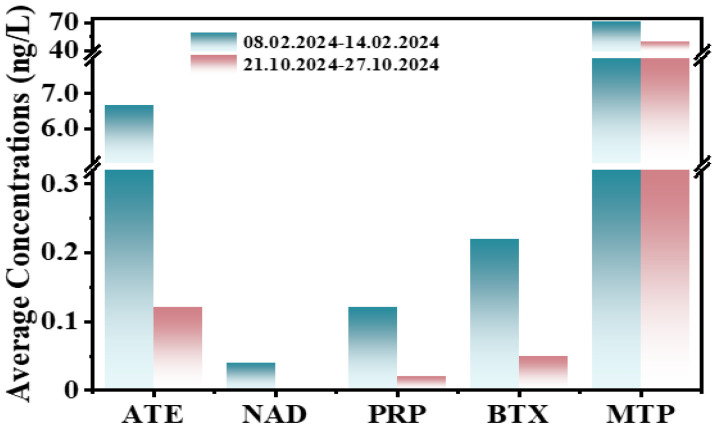
The average values of the beta-blockers found in the monitoring periods. 8–14 February 2024 sampling: ATE, BTX, MTP and PRP—7 samples, NAD—4 samples; 21–27 October 2024 sampling: ATE, MTP, and PRP—7 samples, BTX—4 samples.

Regarding the concentrations measured in the two monitoring periods, it can be observed that the concentrations found in the second sampling are much lower compared to those measured in the first sampling, with the differences in the average values between the two monitoring periods being evident (Figure 1).

It can also be seen that metoprolol is the most detected beta-blocker with an average concentration value of 70.42 ng/L in February and 49.50 ng/L in October, followed by atenolol (6.67 ng/L in February and 0.12 ng/L in October), betaxolol (0.22 ng/L February and 0.05 ng/L October) and propranolol (0.12 ng/L February and 0.02 ng/L in October), respectively. The other beta-blockers studied were found occasionally and in very low concentrations (Table S2).

The concentrations of beta-blockers (Table S2) measured in the two samplings carried out were used to calculate the wastewater mass load, as well as to estimate the consumption of these drugs using wastewater-based epidemiology.

### 2.3. Estimated Beta-Blockers Consumption

**Figure 2:**
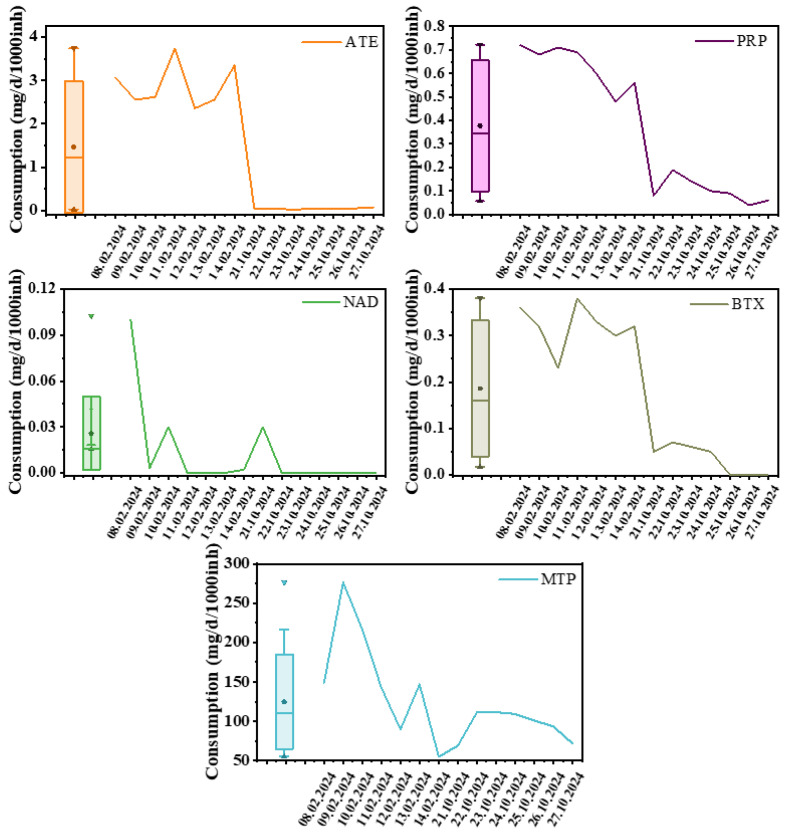
Beta-blockers consumption trend.

The results of our study show a difference in the consumption pattern of the selected pharmaceuticals, depending on the monitoring period, as presented in Figure 2 and Table S3, respectively. The minimum, maximum, and average values calculated for each monitoring period are presented in Table 2.

The highest estimated consumption of the beta-blockers studied was obtained for metoprolol, with values ranging between 54.85 and 276.45 mg/d/1000inh and an average value of 153.70 in February and 95.37 mg/d/1000inh in October 2024.

The estimated consumption of atenolol, with values ranging from 0.03 to 3.74 mg/d/1000inh, which is the second highest after metoprolol, can be explained by the fact that it is a second-generation beta-blocker, used by patients with angina pectoris [5].

Propranolol, which is the third most used beta-blocker in the investigated population with an estimated consumption between 0.04 and 0.72 mg/d/1000inh, is a first-generation beta-blocker used especially in patients suffering from hypertension, angina pectoris and post-myocardial infarction, but which is contraindicated in diabetic patients or those suffering from asthma and chronic obstructive pulmonary disease [5].

Betaxolol, a third-generation beta-blocker, is effective in treating hypertension and coronary heart disease [50]. It recorded daily consumption values between 0.23 and 0.38 mg/d/1000inh in the first sampling, and values between 0.05 and 0.07 mg/d/1000inh in the second sampling, respectively, but quantifiable values were detected in wastewater only on four days.

For the other beta-blockers, such as nadolol, pindolol and acebutolol, consumption could not be estimated, as these compounds were detected in only a few of the samples analyzed (nadolol—five samples, and pindolol and acebutolol—one sample each, out of a total of fourteen samples). The absence of these compounds in the analyzed samples can be explained by the fact that nadolol and pindolol are first-generation beta-blockers and are non-selective, with affinity for β1 and β2 receptors, while acebutolol, which is from the second generation of beta-blockers, has affinity for β1 receptors [5] having the same uses as atenolol and metoprolol, which are the compounds most used in the investigated population.

The estimated consumption expressed in mg/day/1000inh had quite low values, ranging between sub-unit and unit values at most.

It is known that the back-calculation of pharmaceutical use remains a challenge, due to the existence of many parameters that need to be taken into account (population, pharmacokinetic data, source identification—whether the metabolite is endogenous or exogenous, stability of compounds in the sample and in the sewer) [23]. However, the real-time calculation of the number of inhabitants (based on hydrochemical parameters), as well as the application of the correction factor from the literature for the parent compound, reduce the uncertainty of the results. Moreover, no abnormal concentration values were observed in any case during the study, so we believe that direct disposal of the beta-blockers did not occur.

### 2.4. Principal Component Analysis

**Figure 3:**
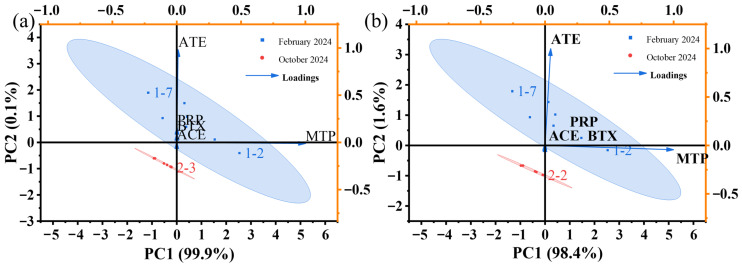
Principal component analysis (PCA) of beta-blockers based on (**a**) wastewater concentrations (ng/L) and (**b**) WBE-estimated consumption (mg/d/1000inh) (“1–7” denotes Day 7 of Week 1).

As shown in Figure 3, PCA was performed on the original (non-standardized) concentration data to preserve the magnitude differences among compounds. The first two principal components explained most of the variance, with PC1 accounting for 98.4% and PC2 contributing 1.6%. The score plot showed that samples were mainly distributed along the PC1 axis.

The loading plot showed that MTP had a strong positive loading on PC1, while ATE (atenolol) was primarily associated with PC2. In contrast, PRP, BTX, and ACE were located near the origin, indicating relatively low contributions to the overall variance.

PCA of the consumption data (Figure 3) showed a similar pattern, with PC1 accounting for 99.9% of the total variance and PC2 contributing only 0.1%. MTP was strongly aligned with PC1, whereas the remaining beta-blockers showed weak loadings and clustered near the origin.

## 3. Discussion

### 3.1. Statistical Approach

The PCA results suggest that temporal variability in wastewater beta-blocker concentrations was largely associated with a single dominant compound, namely MTP. This is consistent with its strong loading on PC1 in both concentration- and consumption-based analyses.

The similarity between the two PCA results indicates that wastewater concentrations largely reflect population-level consumption patterns.

The presence of a minor secondary component in the concentration-based PCA, but not in the consumption-based PCA, suggests that additional processes may influence the measured concentrations in wastewater. These processes likely include dilution, in-sewer transport, and compound-specific fates such as degradation and sorption.

In contrast, PRP, BTX, and ACE showed limited contributions to variance and relatively stable temporal patterns, which may be related to more consistent usage or more stable behavior in the wastewater system.

**Figure 4:**
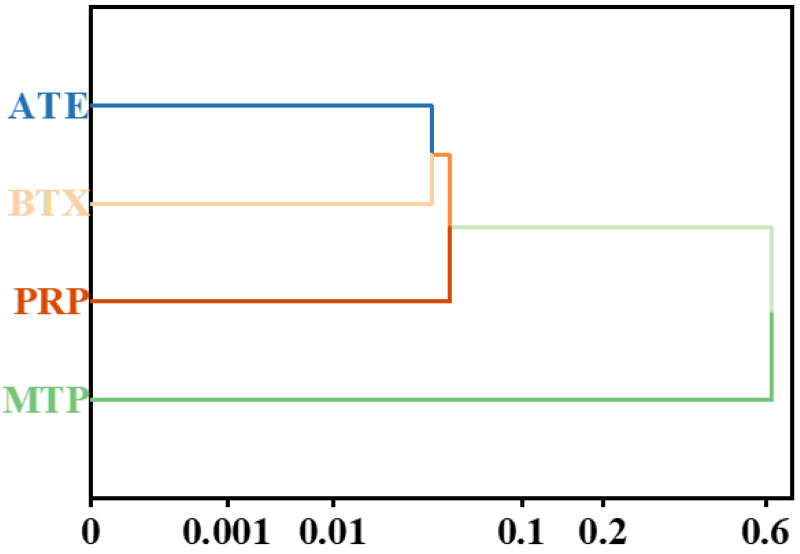
Hierarchical cluster analysis (HCA) of beta-blockers based on wastewater concentrations (ng/L).

Hierarchical cluster analysis (HCA) was applied to group the target beta-blockers according to similarities in their concentration patterns over the sampling period. Prior to analysis, the concentration data were standardized to eliminate the influence of magnitude differences among compounds and to emphasize similarities in temporal variation patterns. As shown in the dendrogram (Figure 4), the compounds are grouped into two main clusters, with ATE, BTX, and PRP forming one cluster, while MTP is clearly separated as an individual cluster, indicating distinct temporal behavior. The x-axis represents the linkage distance (dissimilarity) used in hierarchical clustering, with values ranging from approximately 0 to 0.6, reflecting the degree of dissimilarity between compounds. The relatively small distances among ATE, BTX, and PRP indicate similar temporal patterns, whereas the larger distance separating MTP suggests a markedly different behavior.

The close association among ATE, BTX, and PRP suggests that these beta-blockers exhibit synchronized variations in wastewater concentrations, which is likely related to similar prescription practices and relatively stable consumption within the catchment area. Their comparable temporal profiles imply that population-level usage is a dominant factor governing their occurrence patterns.

By comparison, the pronounced separation of MTP indicates that its temporal variation differs substantially from that of ATE, BTX, and PRP. This distinct behavior may be associated with its higher consumption levels, greater persistence in wastewater, or differences in pharmacokinetic characteristics, such as the relative contributions of parent compounds and metabolites. Consequently, MTP appears to be a major contributor to the overall temporal variability observed in the dataset and may warrant individual consideration in wastewater-based epidemiological assessments.

This phenomenon may be attributed to differences in the consumption patterns of various compounds, as well as their physicochemical properties (e.g., polarity and adsorption behavior) and persistence in wastewater systems. The latter is often reflected by differences in reported wastewater treatment removal efficiencies, which can indirectly influence their temporal dynamics in influent wastewater [51,52].

### 3.2. Comparison with Other Studies

In order to study the consumption behavior of beta-blockers in the investigated population, the results obtained in this study were compared with other results available worldwide. Thus, by analyzing Table 3, it can be clearly seen that, as in our study, metoprolol is the main beta-blocker consumed worldwide. The estimated consumption of metoprolol (54.85–276.45 mg/d/1000inh) in this study is much lower than that in Greece (555.00–590.75 mg/d/1000inh) [53], China (649.80 ± 270.40–1158.90 ± 372.40 mg/d/1000inh) [37], (250.00–260.00 mg/d/1000inh) [54] and England (123.45–413.42 mg/d/1000inh) [21]; higher than in Portugal (11.90–12.90 mg/d/1000inh) [6], Austria (89.00 ± 20.00–92.00 ± 13.00 mg/d/1000inh) [24], Spain (16.00 ± 5.00–34.00 ± 12.00 mg/d/1000inh) [55]; and consistent with that of a suburban area in China (50.00–260.00 mg/d/1000inh) [54]. This estimated consumption could be explained by the fact that metoprolol is part of the second generation of beta-blockers, being widely used in the treatment of hypertension, angina pectoris and heart failure [5].

For atenolol, the estimated consumption (0.03–3.74 mg/d/1000inh) in this study is much lower than that in Greece (502.50–654.25 mg/d/1000inh) [53], Spain (78.00–440.00 mg/d/1000inh) [55,56] and England (333.19–618.59 mg/d/1000inh) [21].

The estimated consumption of propranolol (0.04–0.72 mg/d/1000inh) in this study is lower than that in Portugal (55.60–69.50 mg/d/1000inh) [6], Greece (32.00–93.75 mg/d/1000inh) [53] and England (21,345.86–38,523.02 mg/d/1000inh) [21] while for betaxolol the estimated consumption of (0.05–0.38 mg/d/1000inh) is also lower than that in Greece (2.50–3.50 mg/d/1000inh) [53].

For the other beta-blockers, such as nadolol, pindolol and acebutolol, consumption could not be estimated due to the lack of quantifiable data in the wastewater, nor could it be compared with other values in the literature, as no studies have been found to date; this is the first study focused on the consumption of these drugs.

The high consumption of beta-blocker drugs recorded in the sampling carried out in February 2024, compared to that in October 2024, can be attributed to the cold season, which influences the health of the population. During the cold season, people with cardiovascular problems are more frequently affected by low temperatures, as there is an increase in heart rate to ensure the need for oxygen and maintain a constant body temperature [49]. Furthermore, seasonal differences, together with microbial activity in the sewage system, may produce differences in beta-blocker consumption values [8,37,49].

Moreover, analyzing the data from the literature, a certain typology of beta-blocker use can be observed depending on geographical regions. Thus, the population in Romania most frequently consumes metoprolol, as does the population in Austria (a Central-Eastern European country like Romania) and China. On the other hand, Western European or Mediterranean countries, such as Spain, Portugal, Greece or England, prefer atenolol and propranolol, along with metoprolol.

### 3.3. Uncertainties

The present study primarily considered only parent compounds, without including metabolites, which may lead to a major inconsistency in consumption [33], as parent compounds may originate from human excretion, the dumping of unused pharmaceuticals and pharmaceutical production. Moreover, the difficulty in selecting optimal excretion factors, mainly due to limited pharmacokinetic information, also affects the consumption [23].

Secondly, the population connected to the wastewater treatment plant (WWTP) is another possible source of uncertainty. Population estimation is considered a significant challenge in WBE, as it can introduce errors of between 7 and 55% into the study, depending on how the population connected to the sewer system is calculated (census data, design capacity, hydrochemical parameters) [57]. In this study the hydrochemical parameters were used to determine the population connected to the sewer system, but they can be influenced by industrial and domestic discharges, or weather [25]. Moreover, the use of human biomarkers is being considered to improve how the population is estimated [25] and, finally, the small number of samplings.

Although this study compared variation in beta-blocker use across two monitoring periods, the limited sampling of each period may lead to uncertainty in the data. High temperatures, strong solar radiation, and microbial activity in the sewage system can influence the concentrations of beta-blockers in wastewater. Future sampling over multiple weeks, across different seasons, would help confirm whether the preliminary patterns observed here persist over time.

## 4. Materials and Methods

### 4.1. Chemicals and Reagents

The reference substances of the studied pharmaceuticals, atenolol (ATE), acebutolol (ACE), betaxolol (BTX), nadolol (NAD), metoprolol (MTP), pindolol (PIN) and propranolol (PRP), were purchased from Sigma-Aldrich (Steinheim, Germany). The molecular structure and some physicochemical properties of the studied pharmaceuticals are given in the Supplementary Materials (Table S4).

Stock standard solutions of each individual pharmaceutical at a concentration of 1 mg/mL were prepared in methanol. All solutions were stored at 4 °C in the dark until use.

High-Performance Liquid Chromatography (HPLC) grade methanol (MeOH) and acetonitrile (ACN) were obtained from Merck (Darmstadt, Germany). Ultrapure water was prepared using a Milli-Q ultrapure system, MF-Millipore^TM^ Membrane Filter, 0.45 μm pore size, hydrophilic polyvinylidene fluoride (PVDF) for filtration (Millipore, Burlington, MA, USA). C18-U cartridges 200 mg/3 mL were purchased from Phenomenex (Torrance, CA, USA). For the extraction of the samples, a Resprep Solid Phase Extraction (SPE) system (Restek, Bellefonte, PA, USA) was used.

### 4.2. Instrumentation

The target compounds were analyzed using a high-performance liquid chromatograph Agilent 1200 coupled with a triple Quadrupole spectrometer (QqQ) Agilent 6410 (Santa Clara, CA, USA). A Phenomenex Luna C8(2) column (Torrance, CA, USA) (150 mm × 4.6 mm, 5 μm), thermostated at 35 °C, was used for chromatographic separation. The separation was performed in isocratic mode with a mobile phase composed of ACN:H_2_O (0.1% FA) (23:77, *v*/*v*) for atenolol, nadolol, pindolol, acebutolol and metoprolol, and ACN:H_2_O (0.1% FA) (32:68, *v*/*v*) for propranolol and betaxolol. The mobile phase flow rate was set at 0.3 mL/min, the injection volume was 20 µL, and the analysis time obtained was 9 min. The mass spectrometer was operated in positive mode, using an electrospray voltage of 4000 V. The nebulizer (N_2_) was set to a pressure of 40 psi, a temperature of 350 °C, and a flow rate of 12 L/min. Detection was performed in Multiple Reaction Monitoring (MRM) mode, followed by identification of the precursor molecular ions and the corresponding product ions [21,33].

To determine the optimal parameters, the infusion method was used for each pharmaceutical. Standard solutions were prepared for each pharmaceutical and injected directly into the mass spectrometer. MS/MS transitions were obtained for each pharmaceutical using the most sensitive ions, precursor ions and product ions. For linearity studies, standard mixture solutions were prepared in an ACN:H_2_O mixture (50:50, *v*/*v*) in different incremental amounts, namely, between 1 and 200 ng/mL for atenolol, and between 1 and 100 ng/mL for nadolol, pindolol, acebutolol, metoprolol, propranolol and betaxolol.

The five-point calibration curves were generated as linear functions with a correlation coefficient (R^2^) ≥ 0.9985. The detection limit and the quantification limit were calculated considering the signal-to-noise ratio (S/N) which is equal to 3 and 10, respectively, for each beta-blocker studied [58]. Accuracy showed good values between 91.66% and 102.67%. The performance of the validated LC-MS/MS method is presented in Table S5.

### 4.3. Sample Preparation

Beta-blocking drugs are found in ionized (cationic) form in wastewater, as it has a pH of approximately 7.5. In the cationic form, they are hydrophilic, with a high affinity for water. By adjusting the pH to 10, beta-blocking drugs are in neutral form. In the neutral form, they are lipophilic, with an affinity for organic solvents (methanol) and for adsorbent material (C18) [59].

The optimization of the solid phase extraction of the seven beta-blocking drugs studied was carried out as follows: a volume of 100 mL of distilled water was spiked with 100 μg of each drug (ATE, NAD, PIN, ACE, MTP, PRP, BTX), the distilled water sample being previously alkalinized to pH 10 with 25% ammonium hydroxide. The extraction cartridges were conditioned as follows: 6 mL of distilled water, 6 mL of methanol, and 6 mL of distilled water, after which the synthetic sample (distilled water, alkalized and contaminated with the drugs studied) was passed through them, at a flow rate of approximately 1 mL/min. Subsequently, the cartridges were dried for 20 min under vacuum, and the compounds of interest were eluted with 4 mL of methanol. Each extract was evaporated to dryness under a gentle stream of nitrogen, and the residue was redissolved with 100 μL of acetonitrile for HPLC-PDA analysis. The experiments were performed in triplicate. The HPLC-PDA method used in the analysis of beta-blockers for SPE optimization has been described in [60].

The recovery of the studied beta-blocker drugs using C18-U extraction cartridges (Torrance, CA, USA) varies as follows: atenolol 51.70%, nadolol 94.22%, pindolol 85.40%, acebutolol 89.30%, metoprolol 93.54%, propranolol 88.68%, and betaxolol 91.94% (Table S6). Atenolol exhibits poor retention on the surface of the C18-U extraction cartridge, due to its high polarity (logP 0.16).

The wastewater samples analyzed by LC-MS/MS were subjected to the following extraction procedure. After thawing, the samples were filtered through 0.45 μm membrane filters and alkalized to pH 10 with 25% ammonium hydroxide. Subsequently, a volume of 150 mL of the sample was subjected to the extraction protocol mentioned above, and the residue obtained after extraction and evaporation to dryness under nitrogen was redissolved in 200 μL of the mobile phase for further analysis.

The matrix effect was not measured in this study. We assume that by using extraction cartridges, the matrix effect was eliminated [61,62,63].

### 4.4. Sample Collection

Fourteen composite wastewater samples were collected from the influent of the wastewater treatment plant, which receives wastewater from over 400,000 residents in Cluj-Napoca, Romania and the neighboring communes of Florești, Baciu, Gilău, and Săvădisla. Each day, a composite wastewater sample was obtained by mixing samples collected every hour (1 L each hour) by an automatic device, during a 24 h period, resulting in 24 samples of 1 liter which were further mixed together to obtain a composite sample. Samples were collected in a clean 1 L polypropylene flask, refrigerated, transported at 4 °C, and kept in a freezer until analysis. Therefore, on each sampling day, a composite wastewater sample was collected from the wastewater treatment plant influent, resulting in a total of fourteen samples for analysis.

Seven samples were collected in each sampling period. The samples were collected for seven consecutive days in a conventional week representing “normal” conditions in the investigated seasons (winter and autumn). Each week might reflect baseline consumption [56]. In fact, most of the WBE studies rely on data taken in one week [29,41,42,54,55,56]. The number of inhabitants served by the Cluj-Napoca wastewater treatment plant, as well as pharmacokinetic data for the detected compounds and daily flow rates, were used to back-calculate the consumption of pharmaceutical compounds.

### 4.5. Consumption Estimation Through WBE

The back-calculation of pharmaceutical compound consumption is one of the main challenges in WBE, due to the existence of many parameters that must be considered (population, flow rates, pharmacokinetic data, and in sewer and storage stability) [23]. In our study, each pharmaceutical is used as a biomarker of its own consumption.

The equations to back-calculate the consumption of beta-blockers are as follows according to the WBE methodology [22,26,34,55]:

(1) $$Mass\;load\;(mg/d/1000inh)=\frac{C_{i}\left(\frac{ng}{L}\right)\times Q_{in}\left(\frac{m^{3}}{d}\right)\times 10^{-3}}{\frac{P}{1000}}$$

(2) $$Consumption\;(mg/d/1000inh)=Load\times \frac{1}{{EF}_{i}}\times \frac{{MW}_{par}}{{MW}_{met}}$$

where *C_i_* is the influent concentration of a pharmaceutical; *P* is the number of inhabitants served by the WWTP; *Q_in_* is the mean influent flow of the WWTP; *EF_i_* is the fraction of a given pharmaceutical excreted as the metabolite through urine; *MW_par_* is the molecular weight of the parent pharmaceutical; and *MW_met_* is the molecular weight of the metabolite pharmaceutical.

Normalized daily mass loads (mg/d/1000inh) (Equation (1)) were calculated by multiplying the pharmaceutical concentrations (ng/L) (Table S2) measured over 24 h in the composite wastewater samples by their corresponding wastewater flow rates (m^3^/d) and then normalized by dividing them by the estimated population (Table S1) in the area served by the wastewater treatment plant from which the samples were derived. Pharmaceutical consumption (Equation (2)) was estimated by multiplying the normalized daily mass values by the fraction of a given drug excreted as a metabolite in urine and the ratio of the molecular weight of the parent drug (*MW_par_*) to the molecular weight of the metabolite drug (*MW_met_*) (Table S3). The parent compounds are considered biomarkers (human metabolic products). Thus, for the pharmaceuticals studied, the excretion rates considered are as follows: atenolol 50%, pindolol 80%, nadolol 24.6%, acebutolol 40%, propranolol 4%, betaxolol 15%, and metoprolol 10% [64,65].

The population size estimate was made based on hydrochemical parameters (chemical oxygen demand (COD), biological oxygen demand (BOD), total nitrogen (N), ammonium nitrogen (NH_4_-N) or total phosphorus (P)), and the information is presented in the following study [36] and in Table S1.

Been et al. [66] and Rico et al. [67] proposed the use of ammonium nitrogen for population estimation, as it occurs in wastewater, mainly as a result of urea hydrolysis, and is specific to urine. Ammonium nitrogen can be successfully used for population estimation [30,66], being sensitive to population movements (tourism, commuters) [68], but only during periods without precipitation, because this causes the dilution of ammonia nitrogen in wastewater [66].

In this study, beta-blocker consumption was investigated using the WBE approach in two periods in Cluj-Napoca, Romania, but this research has some uncertainties.

### 4.6. Statistical Analysis

Principal component analysis (PCA) and hierarchical cluster analysis (HCA) were conducted using both wastewater concentrations of beta-blockers (ng/L) and wastewater-based epidemiology (WBE)-estimated consumption data (mg/d/1000inh) to examine temporal variability from environmental and population-level perspectives.

## 5. Conclusions

This study is the first attempt in Romania to investigate the consumption of beta-blockers (atenolol, acebutolol, betaxolol, metoprolol, nadolol, pindolol, and propranolol) among the urban population using wastewater-based epidemiology.

For this purpose, specific and sensitive methods have been developed to allow the analysis of selected pharmaceuticals at the trace levels present in wastewater, such as solid phase extraction and liquid chromatography coupled with tandem mass spectrometry.

Even though the WBE methodology is accompanied by a series of uncertainties related to the choice of a specific metabolite or the accurate determination of the number of inhabitants connected to a sewage system, this methodology can provide rapid information related to the consumption of selected pharmaceutical products, as well as an overview of the consumption behavior of the population during sensitive periods from a public health point of view.

For the present study, the results of beta-blocker determinations in wastewater show significant variations between the two monitoring periods, with higher values in the sampling conducted in the cold season (February) compared to the sampling in the moderate temperature season (October). This may be due to the fact that low ambient temperatures increase the consumption of beta-blockers, due to the additional effort made by the cardiovascular system to supply oxygen and maintain a constant body temperature.

The estimated consumption of beta-blockers among the investigated urban population recorded the highest values for metoprolol, with values between 54.85 and 276.45 mg/d/1000inh and an average value of 153.70 in February and 95.37 mg/d/1000inh in October 2024.

Nadolol, pindolol and acebutolol were detected in only a few of the samples analyzed, which is why their consumption could not be estimated. The absence of these compounds in the analyzed wastewater samples can be partly explained by their non-selective effect (nadolol and pindolol) or by the existence of other, more popular beta-blockers, such as atenolol and metoprolol as alternatives to acebutolol.

By comparing the estimated consumption in various studies worldwide, a certain typology of beta-blocker use can be observed that can be correlated with different geographical areas. Thus, while in Romania, Austria and China metoprolol is most frequently consumed, Western European or Mediterranean countries, such as Spain, Portugal, Greece or England, prefer atenolol and propranolol, along with metoprolol. This typology can most likely be explained by the role that a country has in a certain region in the production and distribution of pharmaceuticals, as well as by the existing trade links between the countries.

## Figures and Tables

**Table 1:** The minimum and maximum values of selected beta-blockers in the sewage of Cluj-Napoca.

Concentrations of Beta-Blockers (ng/L)				
Compound	February 2024 (n = 7)		October 2024 (n = 7)	
	Minimum	Maximum	Minimum	Maximum
ATE	5.76	8.28	0.09	0.21
NAD	n.d.	0.12	n.d.	0.03
PIN	n.d.	0.004	n.d.	n.d.
ACE	n.d.	0.25	n.d.	n.d.
PRP	0.09	0.13	0.01	0.04
BTX	0.15	0.25	n.d.	0.06
MTP	25.51	124.59	35.01	57.84

n.d.—not detected. 8–14 February 2024 sampling: ATE, BTX, MTP and PRP—7 samples, NAD—4 samples, ACE and PIN—1 sample. 21–27 October 2024 sampling: ATE, MTP, and PRP—7 samples, BTX—4 samples, NAD—1 sample, ACE and PIN—not detected.

**Table 2:** The minimum, maximum, and average values of the consumption of the selected beta-blockers for monitoring periods.

Consumption (mg/d/1000inh)						
Compound	February 2024 (n = 7)			October 2024 (n = 7)		
	Minimum	Maximum	Average	Minimum	Maximum	Average
ATE	2.36	3.74	2.89	0.03	0.08	0.05
NAD	n.a.	0.10	0.02	n.a.	0.03	n.a.
PIN	n.a.	0.001	n.a.	n.a.	n.a.	n.a.
ACE	n.a.	0.13	n.a.	n.a.	n.a.	n.a.
PRP	0.48	0.72	0.63	0.04	0.19	0.10
BTX	0.23	0.38	0.32	n.a.	0.07	0.03
MTP	54.85	276.45	153.70	68.93	111.65	95.37

n.a.—not applicable. 8–14 February 2024 sampling: ATE, BTX, MTP and PRP—7 samples, NAD—4 samples, ACE and PIN—1 sample. 21–27 October 2024 sampling: ATE, MTP, and PRP—7 samples, BTX—4 samples, NAD—1 sample, ACE and PIN—not detected.

**Table 3:** Estimated consumption of the studied beta-blocker drugs expressed as mg/d/1000inh from different countries.

Country	Year	WWTP	ATE	NAD	PIN	ACE	PRP	BTX	MTP	Ref.
Portugal	2019	Ponte de Moreira’s	–	–	–	–	(R) 55.60	–	(R) 12.90	[6]
							(S) 69.50		(S) 11.90	
England	2023	South-West England	333.19–618.59	–	–	–	21,345.86–38,523.02	–	123.45–413.42	[21]
Austria	2016–2020	Innsbruck	–	–	–	–	–	–	89.00 ± 20.00	[24]
	2020		–	–	–	–	–	–	92.00 ± 13.00	
China	2020–2021	China	**MTP**	North: 1158.90 ± 372.40; East: 996.50 ± 384.50; North-East: 996.40 ± 297.60; South: 888.70 ± 469.40; North-West: 886.00 ± 303.30; Central China: 748.40 ± 350.20; South-West: 649.80 ± 270.40						[37]
Greece	2019	Athens	654.25	–	–	–	93.75	2.50	555.00	[53]
	2020		502.50	–	–	–	32.00	3.50	590.75	
China	2023	Suburban	4.80–15.00	–	–	–	–	–	50.00–200.00	[54]
		Urban	14.50–15.50	–	–	–	–	–	250.00–260.00	
Spain	2021	Puerto Real	192.00 ± 123.00	–	–	–	–	–	31.00 ± 10.00	[55]
		Jerez de la Frontera	128.00 ± 46.00	–	–	–	–	–	16.00 ± 5.00	
		Cadiz	112.00 ± 44.00	–	–	–	–	–	34.00 ± 12.00	
Spain	2022	Madrid, Tarragona, Reus	78.00–440.00	–	–	–	–	–	–	[56]
Romania	2024 (February)	Cluj-Napoca	2.36–3.74	n.d.–0.10	n.d.–0.001	n.d.–0.13	0.48–0.72	0.23–0.38	54.85–276.45	This study.
	2024 (October)		0.03–0.08	n.d.–0.03	n.d.	n.d.	0.04–0.19	0.05–0.07	68.93–111.65	

– —not applicable; n.d.—not detected; (R)—right isomer; (S)—left isomer. Note: standard deviations were presented only if they were published in the cited article.

## Data Availability

Data are contained within the article and Supplementary Materials.

## Supplementary Materials

The following supporting information can be downloaded at: https://www.mdpi.com/article/10.3390/molecules31081249/s1, Table S1: The flow rates at the inlet of the wastewater treatment plant (L/s), the concentrations of COD, BOD, P and NH_4_-N (mg/L) provided by the wastewater treatment plant of Cluj-Napoca, as well as the population calculated based on the concentration of hydrochemical parameters for February 2024 and October 2024 [36]; Table S2: Concentrations (ng/L) of beta-blockers studied in wastewater samples; Table S3: The estimated consumption of beta-blocker drugs studied; Table S4: The molecular structure and some physico-chemical properties of the studied pharmaceuticals; Table S5: Optimized collision energies, and ions (*m*/*z*) of the targeted pharmaceuticals, the retention time (RT), linearity, and limit of detection and quantification (LOD, LOQ); Table S6: Recovery of the studied beta-blockers by the HPLC-PDA method.

## Abbreviations

The following abbreviations are used in this manuscript:

WBE	Wastewater-based epidemiology
inh	Inhitants
n	Number of Samples
COD	Chemical Oxygen Demand
BOD	Biological Oxygen Demand
N	Total Nitrogen
NH_4_-N	Ammonium Nitrogen
P	Total Phosphorus
ATE	Atenolol
NAD	Nadolol
PIN	Pindolol
ACE	Acebutolol
PRP	Propranolol
BTX	Betaxolol
MTP	Metoprolol
n.a.	not applicable
n.d.	Not Detected
*MW_par_*	the Molecular Weight of the Parent Drug
*MW_met_*	the Molecular Weight of the Metabolite Drug
PCA	Principal Component Analysis
PC	Principal Component
HCA	Hierarchical Cluster Analysis
Ref	Reference
WWTP	Wastewater Treatment Plant
HPLC	High-Performance Liquid Chromatography
MeOH	Methanol
ACN	Acetonitrile
PVDF	Polyvinylidene Fluoride
SPE	Solid Phase Extraction
USA	United States of America
QqQ	Triple Quadrupole Spectrometer
FA	Formic Acid
MRM	Multiple Reaction Monitoring
MS/MS	Tandem Mass Spectrometry
LOD	Limit of Detection and Quantification
LOQ	Limit of Detection and Quantification
S/N	Signal-to-Noise Ratio
LC-MS/MS	Liquid Chromatography coupled with tandem Mass Spectrometry
HPLC-PDA	High-Performance Liquid Chromatography with Photodiode
*Q_in_*	the Mean Influent Flow of the WWTP
*EF_i_*	the Fraction of a Given Pharmaceutical Excreted as the Metabolite through Urine
