# Supplementary material for: Estimation of Beta-Blocker Consumption in the Urban Population Using the Wastewater-Based Epidemiology Approach
Source: Molecules. 2026 Apr 9;31(8):1249. doi: 10.3390/molecules31081249 (PMC13119527; doi:10.3390/molecules31081249)
Supplement: Supplementary file 1 [file molecules-31-01249-s001.zip › molecules-4174782-supplementary.pdf]

SUPPLEMENTARY INFORMATION

Article

Estimation of beta-blocker consumption in the urban population using the wastewater based epidemiology approach

Mihail Simion Beldean-Galea[1,2*], Mihaela-Cătălina Herghelegiu[1,2*], Ye Yang[1], Robert Tötös[3], Maria Concetta Bruzzoniti[4], Ioana Elena Beldean-Galea[5], Sorina Irimie[5], Anda Curta[5]

[1] Faculty of Environmental Science and Engineering, Babeș-Bolyai University, 30 Fântânele Str., RO-400294 Cluj-Napoca, Romania;
[2] "Raluca Ripan" Institute for Research in Chemistry, Babeș-Bolyai University, 30 Fântânele Str., RO-400294 Cluj-Napoca, Romania;
[3] Faculty of Chemistry and Chemical Engineering, Babeş-Bolyai University, 11 Árány Janos Str., RO-400028 Cluj-Napoca, Romania;
[4] Department of Chemistry, University of Turin, Via P. Giuria 5, 10125 Turin, Italy;
[5] National Institute of Public Health, Regional Centre of Public Health Cluj, 6 Pasteur Str., RO-400349, Cluj-Napoca, Romania;
* Correspondence: simion.beldean@ubbcluj.ro (M.S.B.-G.); mihaela.herghelegiu@ubbcluj.ro (M.-C.H.)

The supplementary information contains 8 pages, and includes 6 tables and 0 figures.

List of contents of the supplementary information:

Table S1. The flow rates at the inlet of the wastewater treatment plant (L/s), the concentrations of COD, BOD, P and NH_4-N (mg/L) provided by the wastewater treatment plant of Cluj-Napoca, as well as the population calculated based on the concentration of hydrochemical parameters for February 2024 and October 2024 [36].

Table S2. Concentrations (ng/L) of beta-blockers studied in wastewater samples.

Table S3. The estimated consumption of beta-blocker drugs studied.

Table S4. The molecular structure and some physico-chemical properties of the studied pharmaceuticals.

Table S5. Optimized collision energies, and ions (*m/z*) of the targeted pharmaceuticals, the retention time (RT), linearity, and limit of detection and quantification (LOD, LOQ).

Table S6. Recovery of the studied beta-blockers by the HPLC-PDA method.

Table S1. The flow rates at the inlet of the wastewater treatment plant (L/s), the concentrations of COD, BOD, P and NH_4-N (mg/L) provided by the wastewater treatment plant of Cluj-Napoca, as well as the population calculated based on the concentration of hydrochemical parameters for February 2024 and October 2024 [36].

Day	Flow (L/s)	COD (mg/L)	Population based on COD	BOD (mg/L)	Population based on BOD	P (mg/L)	Population based on P	NH_4-N (mg/L)	Population based on NH_4-N
				February 2024					
**08.02.2024**	1,079.00	395.50	288,053	–	–	–	–	–	–
**09.02.2024**	1,128.00	389.50	296,565	–	–	–	–	–	–
**10.02.2024**	1,132.00	216.50	165,428	–	–	–	–	–	–
**11.02.2024**	1,149.00	308.20	239,032	–	–	–	–	–	–
**12.02.2024**	1,036.00	346.20	242,098	144.20	215,123	6.35	334,348	41.71	439,233
**13.02.2024**	1,093.00	636.00	469,225	246.90	388,601	–	–	–	–
**14.02.2024**	1,093.00	365.40	269,583	–	–	–	–	–	–
**Average**			**281,426**		**301,862**		**334,348**		**439,233**
				October 2024					
**21.10.2024**	1,212.96	445.60	364,835	214.40	374,485	6.84	421,666	43.17	532,261
**22.10.2024**	1,187.50	370.00	296,578	185.60	317,376	–	–	–	–
**23.10.2024**	1,192.13	350.20	281,802	–	–	–	–	–	–
**24.10.2024**	1,164.35	351.60	276,336	–	–	–	–	–	–
**25.10.2024**	1,215.28	363.90	298,512	–	–	–	–	–	–
**26.10.2024**	1,157.41	330.00	257,813	–	–	–	–	–	–
**27.10.2024**	1,187.50	282.90	226,762	–	–	–	–	–	–
**Average**			**286,091**		**345,931**		**421,666**		**532,261**

[36] Beldean-Galea, M.S.; Herghelegiu, M.-C.; Combès, A.; Vial, J.; Totos, R.; Bruzzoniti, M.C.; Coman, M.-V. A surveillance of paracetamol and nonsteroidal anti-inflammatory drug consumption in Cluj-Napoca, Romania, using wastewater-based epidemiology. *Metabolites* **2025**; *15*, 576.

COD – chemical oxygen demand; BOD – biological oxygen demand; NH_4-N – ammonium nitrogen; P – total phosphorus.

Table S2. Concentrations (ng/L) of beta-blockers studied in wastewater samples.

**Day**	**Concentrations of beta-blockers (ng/L)**						
	**ATE**	**NAD**	**PIN**	**ACE**	**PRP**	**BTX**	**MTP**
February 2024							
08.02.2024	7.22	0.12	0.004	0.25	0.13	0.25	70.18
09.02.2024	5.76	0.003	n.d.	n.d.	0.12	0.21	124.59
10.02.2024	5.89	0.03	n.d.	n.d.	0.13	0.15	97.20
11.02.2024	8.28	n.d.	n.d.	n.d.	0.12	0.25	63.07
12.02.2024	5.79	n.d.	n.d.	n.d.	0.12	0.24	44.00
13.02.2024	5.95	n.d.	n.d.	n.d.	0.09	0.21	68.37
14.02.2024	7.79	0.002	n.d.	n.d.	0.11	0.22	25.51
Average	6.67	0.04	–	–	0.12	0.22	70.42
October 2024							
21.10.2024	0.10	0.03	n.d.	n.d.	0.02	0.04	35.01
22.10.2024	0.09	n.d.	n.d.	n.d.	0.04	0.06	57.80
23.10.2024	0.09	n.d.	n.d.	n.d.	0.03	0.05	57.70
24.10.2024	0.10	n.d.	n.d.	n.d.	0.02	0.04	57.84
25.10.2024	0.13	n.d.	n.d.	n.d.	0.02	n.d.	51.17
26.10.2024	0.13	n.d.	n.d.	n.d.	0.01	n.d.	49.70
27.10.2024	0.21	n.d.	n.d.	n.d.	0.01	n.d.	37.31
Average	0.12	–	–	–	0.02	0.05	49.50

n.d. – not detected; – —not calculated

ATE – Atenolol, NAD – Nadolol, PIN – Pindolol; ACE – Acebutolol; PRP – Propranolol; BTX – Betaxolol; MTP – Metoprolol.

Table S3. The estimated consumption of beta-blocker drugs studied.

Day	Consumption (mg/d/1000inh)						
	**ATE**	**NAD**	**PIN**	**ACE**	**PRP**	**BTX**	**MTP**
February 2024							
08.02.2024	3.07	0.10	0.001	0.13	0.72	0.36	148.95
09.02.2024	2.56	0.003	n.a.	n.a.	0.68	0.32	276.45
10.02.2024	2.62	0.03	n.a.	n.a.	0.71	0.23	216.44
11.02.2024	3.74	n.a.	n.a.	n.a.	0.69	0.38	142.54
12.02.2024	2.36	n.a.	n.a.	n.a.	0.60	0.33	89.67
13.02.2024	2.56	n.a.	n.a.	n.a.	0.48	0.30	146.99
14.02.2024	3.35	0.002	n.a.	n.a.	0.56	0.32	54.85
October 2024							
21.10.2024	0.04	0.03	n.a.	n.a.	0.08	0.05	68.93
22.10.2024	0.04	n.a.	n.a.	n.a.	0.19	0.07	111.41
23.10.2024	0.03	n.a.	n.a.	n.a.	0.14	0.06	111.65
24.10.2024	0.04	n.a.	n.a.	n.a.	0.10	0.05	109.33
25.10.2024	0.05	n.a.	n.a.	n.a.	0.09	n.a.	100.94
26.10.2024	0.05	n.a.	n.a.	n.a.	0.04	n.a.	93.38
27.10.2024	0.08	n.a.	n.a.	n.a.	0.06	n.a.	71.92

n.a. - not applicable.

ATE – Atenolol, NAD – Nadolol, PIN – Pindolol; ACE – Acebutolol; PRP – Propranolol; BTX – Betaxolol; MTP – Metoprolol.

Table S4. The molecular structure and some physico-chemical properties of the studied pharmaceuticals.

Beta-blocker	Molecular structure	Chemical formula	Molecular mass (amu)	LogP	pKa
Atenolol	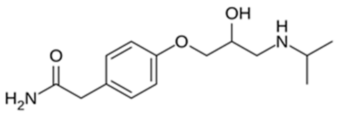	$C_{14}H_{22}N_2O_3$	266.34	0.16	9.58
Nadolol	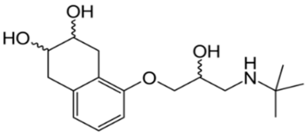	$C_{17}H_{27}NO_4$	309.4	0.81	9.67
Pindolol	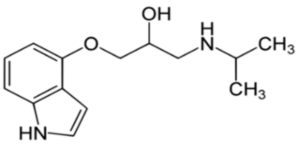	$C_{14}H_{20}N_2O_2$	248.32	1.75	9.54
Acebutolol	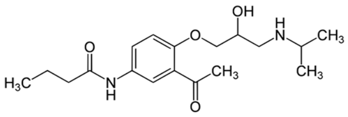	$C_{18}H_{28}N_2O_4$	336.40	1.71	9.52
Propranolol	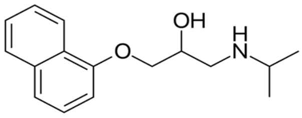	$C_{16}H_{21}NO_2$	259.34	3.48	9.53
Betaxolol	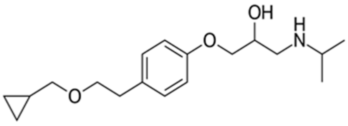	$C_{18}H_{29}NO_3$	307.40	2.81	9.21
Metoprolol	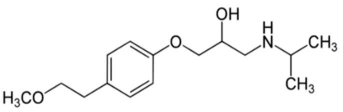	$C_{15}H_{25}NO_3$	267.36	2.15	9.56

Physicochemical properties (Molecular mass, LogP, pKa) from PubChem databases. https://pubchem.ncbi.nlm.nih.gov/ (accessed on 24 November 2025).

Table S5. Optimized collision energies, and ions (*m/z*) of the targeted pharmaceuticals, the retention time (RT), linearity, and limit of detection and quantification (LOD, LOQ).

Beta-blocker	RT (min)	Precursor Ion (*m/z*)	Product Ions (*m/z*)	Fragmentor (V)	Collision energy (eV)	Calibration curve equation	LOD (ng/mL)	LOQ (ng/mL)	R^2	Accuracy (%)
ATE	3.99	267.6	190.3<br>145.3	120	20	y = 314.54x+56.02	0.08	0.15	0.9993	102.50
NAD	4.36	310.5	254.4<br>236.4	120	12	y = 725.34x+123.53	0.19	0.37	0.9992	100.39
PIN	4.96	249.4	172.4<br>116.4	100	10	y = 1,175.27x+245.57	0.22	0.43	0.9985	91.66
ACE	5.50	337.5	319.5<br>116.3	120	20	y = 901.06x+299.88	0.11	0.21	0.9985	101.46
PRP	5.56	260.5	183.4<br>116.4	110	20	y = 600.99x+66.09	0.13	0.26	0.9995	102.67
BTX	5.86	308.5	133.4<br>116.4	110	20	y = 240.45x+6.49	0.22	0.44	0.9991	101.77
MTP	6.23	268.6	159.3<br>116.3	120	20	y = 387.37x+28.21	0.19	0.37	0.9999	98.72

ATE – Atenolol, NAD – Nadolol, PIN – Pindolol; ACE – Acebutolol; PRP – Propranolol; BTX – Betaxolol; MTP – Metoprolol.

RT – Retention time; LOD – Limit of detection; LOQ – Limit of quantification; R^2 – correlation coefficient.

Table S6. Recovery of the studied beta-blockers by the HPLC-PDA method.

Beta-blockers	Quantity (µg)			Mean recovery (%)	RSD (%)
	Initial	Added	Found		
ATE	0	100	51.88	51.70	0.71
	0	100	51.61		
	0	100	51.62		
NAD	0	100	95.07	94.22	0.80
	0	100	93.91		
	0	100	93.67		
PIN	0	100	86.37	85.40	0.99
	0	100	84.81		
	0	100	85.04		
ACE	0	100	91.70	89.30	2.52
	0	100	88.97		
	0	100	87.24		
MTP	0	100	94.74	93.54	1.17
	0	100	93.27		
	0	100	92.60		
PRP	0	100	90.77	88.68	2.05
	0	100	87.69		
	0	100	87.57		
BTX	0	100	93.41	91.94	1.39
	0	100	91.35		
	0	100	91.07		

ATE – Atenolol, NAD – Nadolol, PIN – Pindolol; ACE – Acebutolol; PRP – Propranolol; BTX – Betaxolol; MTP – Metoprolol.

RSD –Relative standard deviation.
